# In vitro evaluation and molecular docking of QS-21 and quillaic acid from *Quillaja saponaria* Molina as gastric cancer agents

**DOI:** 10.1038/s41598-020-67442-3

**Published:** 2020-06-29

**Authors:** Leda Guzmán, Katherine Villalón, María José Marchant, María Elena Tarnok, Pilar Cárdenas, Gisela Aquea, Waldo Acevedo, Leandro Padilla, Giuliano Bernal, Aurora Molinari, Alejandro Corvalán

**Affiliations:** 1 Laboratorio de Química Biológica, Instituto de Química, Facultad de Ciencias, Pontificia Universidad Católica de Valparaíso, Valparaíso, Chile; 2 Natural Response S.A, Av. Industrial 1970, Quilpué, Región de Valparaíso Chile; 3 Laboratorio de Biología Molecular y Celular del Cáncer, Departamento de Ciencias Biomédicas, Facultad de Medicina, Universidad Católica del Norte, Coquimbo, Chile; 4 Laboratorio de Química Orgánica, Instituto de Química, Facultad de Ciencias, Pontificia Universidad Católica de Valparaíso, Valparaíso, Chile; 5 Laboratorio de Oncología, Departamento Hematología y Oncología, Facultad de Medicina, Pontificia Universidad Católica de Chile, Santiago, Chile; 6 Advanced Center for Chronic Diseases, Santiago, Chile

**Keywords:** Computational biology and bioinformatics, Cancer, Cancer therapy, Gastrointestinal cancer, Chemical biology, Natural products

## Abstract

The cytotoxic mechanism of the saponin QS-21 and its aglycone quillaic acid (QA) was studied on human gastric cancer cells (SNU1 and KATO III). Both compounds showed in vitro cytotoxic activity with IC_50_ values: 7.1 μM (QS-21) and 13.6 μM (QA) on SNU1 cells; 7.4 μM (QS-21) and 67 μM (QA) on KATO III cells. QS-21 and QA induce apoptosis on SNU1 and KATO III, as demonstrated by TUNEL, Annexin-V and Caspase Assays. Additionally, we performed in silico docking studies simulating the binding of both triterpenic compounds to key proteins involved in apoptotic pathways. The binding energies (∆*G*_*bin*_) thus calculated, suggest that the pro-apoptotic protein Bid might be a plausible target involved in the apoptotic effect of both triterpenic compounds. Although QA shows some antiproliferative effects on SNU1 cells cultured in vitro, our results suggest that QS-21 is a more powerful antitumor agent*,* which merits further investigation regarding their properties as potential therapeutic agents for gastric cancer.

## Introduction

**Figure 1:**
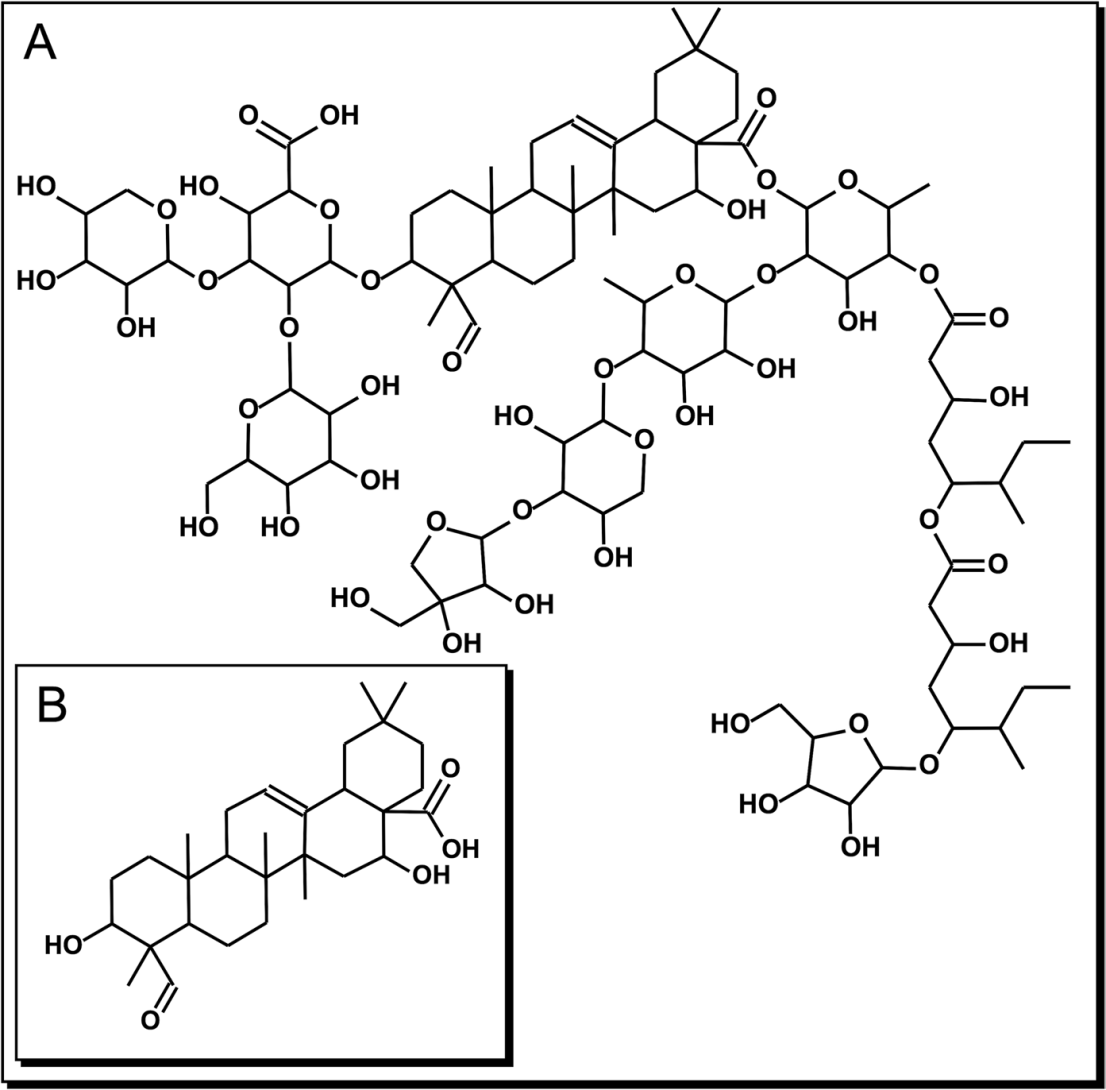
Chemical structure of the triterpenic compounds employed in this study. (**A**): QS-21: C_92_H_148_O_46_ and (**B**) QA: C_30_H_46_O_5_.

Gastric cancer (GC) is the third deadliest tumor malignancy worldwide. Despite an overall decline in incidence over the last decades, GC remains the fifth most common type of cancer^1^. Unfortunately, multidrug resistance has adversely impacted the effectiveness of many forms of chemotherapy^2^. One way to increase the effectiveness of chemotherapy is the synergistic combination of traditional anticancer drugs with plant compounds (phytochemicals)^3^; an even more promising approach is the use of phytochemicals as antitumor agents instead of traditional anticancer drugs, in order to reduce the adverse effects related to those drugs-toxicity in normal tissues and drug resistance. A remarkable group of phytochemicals are saponins, a widespread family of natural glycosides of steroid or triterpenoid aglycones^4^. The bark of *Quillaja saponaria* Molina (QS)—an evergreen tree native from Chile—is widely used as a source of physiologically active saponins based on the triterpenic aglycone quillaic acid (QA). The latter is a pentacyclic triterpenoid, i.e. olean-12-ene substituted by hydroxy groups at positions 3 and 16, an oxo group at position 23 and a carboxyl group at position 28. The saponins of *Q. saponaria* are highly diverse in structure; a remarkable member of that family of compounds is QS-21^6^. QS-21 is an acylated 3,28-bidesmodic triterpene glycosides of the aglycone QA, as shown in Fig. 1. QA exhibits diverse biological activities, such as hemolytic, anti-inflammatory, immune-stimulatory, antinociceptive, antiviral and cytotoxic^4–6^. Most studies on biological effects of QS-21 have been focused on the immune response, however, the cytotoxic and anti-proliferative effects both QS-21 and QA in GC human cell lines have not yet been studied^6,7^. On the other hand, Wang et al.^7^ demonstrated the in vitro antitumor activity of QS-21 on 11 tumor cell lines as melanoma, breast cancer, small cell lung cancer, prostate cancer.

The purpose of this study was to determine the in vitro therapeutic potential and the cytotoxic mechanism of saponin QS-21 and its aglycone QA on two cell lines of human GC (SNU1 and KATO III). To complement the in vitro study and to attain insight on the mechanism of action of QS-21 and QA to trigger cell death, we performed molecular docking studies of these compounds with known 3D structures of the proteins involved in cell death signaling, such as Death Receptor (DR4), FAS-protein and C-Jun-N-terminal Kinase (JNK1), RAC-alpha serine/threonine-protein kinase (AKT1), pro-apoptotic protein BAX, BID, and fibroblast growth factor receptor 2 (FGFR-2); all of these proteins are key components in the extrinsic apoptotic pathway and overexpressed in solid tumors cell^8–10^.

## Results

### Cytotoxicity assay of QS-21 and QA on gastric cancer cells (MTS and LDH assays)

In order to determine the therapeutic potential of QS-21 and QA on the treatment of gastric cancer, we first tested their impact on the viability of the in vitro cell lines KATO III, SNU1 and GES-1, using the MTS test^11^.

**Figure 2:**
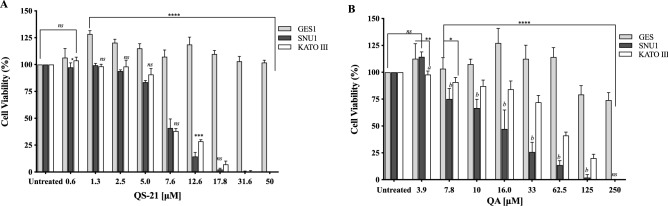
Effect of QS-21 and QA on cell viability by MTS assay. Data expressed as means ± SD of five independent experiments. (**A**) ****P < 0.0001 when SNU1 and KATO III were compared with GES-1, ***P < 0.005 SNU1 versus KATO III, *P < 0.05 GES-1 versus SNU1 and *ns* (no significant) between the groups treated. (**B**) ****P < 0.0001 when SNU1 and KATO III were compared with GES-1, ** and *indicated *P* < 0.01 and *P* < 0.05 GES-1 versus KATO III, *P* < 0.005 (a and b) KATO III versus SNU1.

All the cell types under study were treated with QS-21 and QA at different concentration ranges, during 24 h and Staurosporine (STS) (see Material and Methods and Supplementary Fig. S1). QA and QS-21 were cytotoxic to KATO III and SNU1 tumor lines in a dose dependent way (Fig. 2A, B); both compounds were barely cytotoxic on GES-1 cells, a human normal cell line employed as control (Fig. 2A, B). QA showed a cytotoxic activity, which was more pronounced on SNU1 than KATO III cells—IC_50_ value of 13.6 µM and 67 µM, respectively (Fig. 2B, Table 1). It should also be noted that SNU1 was more sensitive to concentrations over 16.0 µM of QA. In the tested conditions, QS-21 showed a stronger cytotoxic effect than QA on both cancer cell lines under study, with IC_50_ values of 7.1 μM and 7.4 μM respectively.

**Table 1:** IC_50_ values of QS-21 and QA in gastric cancer cell lines and gastric human normal cell lines.

Cell lines	QS-21	QA
IC_50_ (µM) ± SD		
SNU1	7.1 (± 0.4)	13.6 (+ 3.7)
KATO III	7.4 (± 1.4)	67.0 (± 17.9)
GES-1	> 100	> 100

The IC_50_ values represent the mean of at least 5 independent experiments (SD).

**Figure 3:**
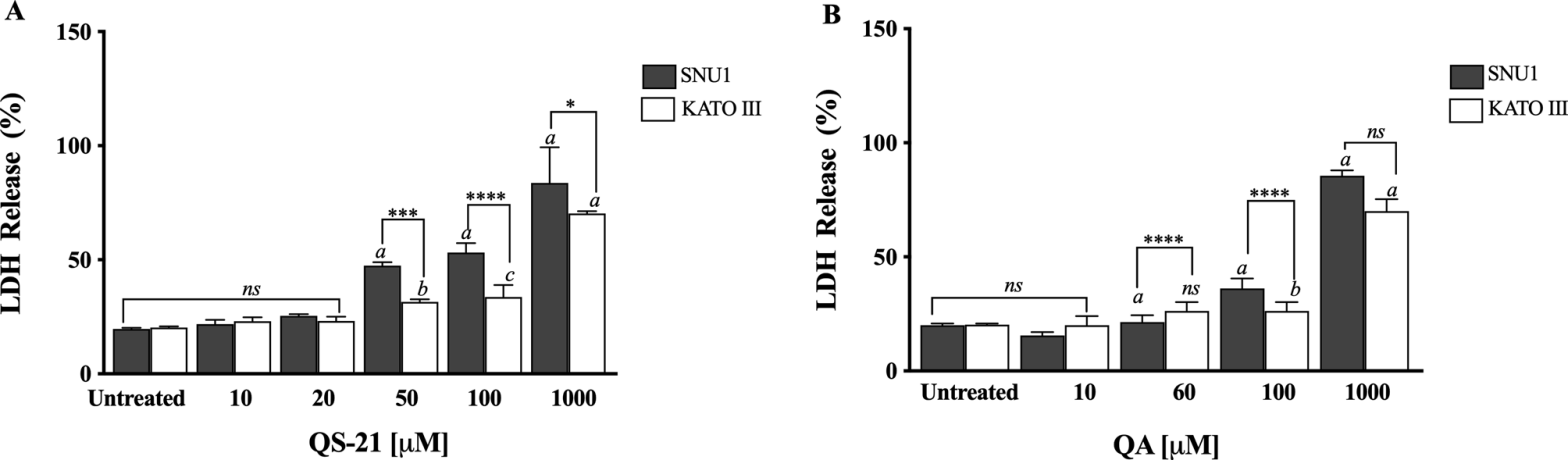
Analysis of LDH activity. Results are expressed as percentage of LDH release minus the vehicle control value. a, b and c indicate a *P* < 0.0001, *P* < 0.01 and *P* < 0.05, respectively when is compared SNU1 and KATO III to untreated cells, ****, *** and * indicated a *P* < 0.0001, *P* < 0.005 and *P* < 0.05 between SNU1 versus KATO III treated with QS-21 (**A**) or QA (**B**) and *ns* (no significant) between cells lines. Data points are mean values ± SD from three independent experiments, each performed in triplicate.

**Figure 4:**
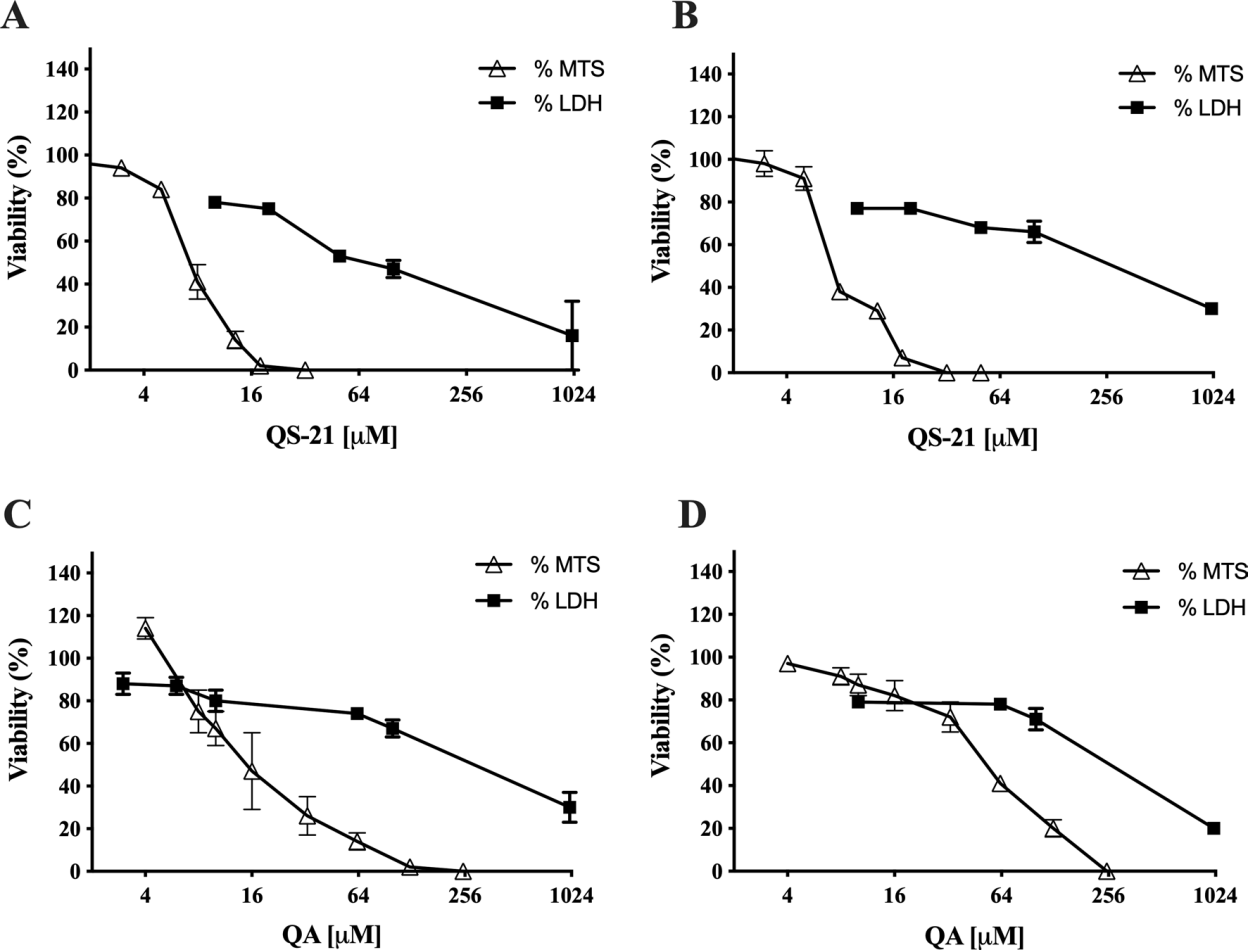
Correlation between MTS and LDH assay. Comparison of effect of QS-21 and QA on the metabolic viability (determined by MTS) and the viability in terms of membrane integrity (determined by release of LDH) of SNU1 and KATO III cells. (**A**) and (**B**) QS-21 on SNU1 and KATO III, respectively. (**C**) and (**D**) QA on SNU1 and KATO III, respectively.

QS-21 and QA are surfactant molecules and under the conditions employed in the in vitro tests, both compounds may interact with lipid components of biological membranes leading to the formation of pores or even inducing the partial or complete rupture of the membranes. Also, some molecules can generate the disruption of the integrity of lipid rafts, resulting in an apoptotic or pro-apoptotic signal as methyl-β-cyclodextrin or Avicin D^12,13^. In order to determine if the loss on cell viability detected by MTS method in SNU1 and KATO III cells after exposure to QS-21 and QA was just a consequence the disruption of plasma membrane, or a different mechanism was involved, we measured the release of the cytosolic of lactate dehydrogenase enzyme (LDH) to the extracellular medium upon exposure of the cells to both triterpenic compounds. For this purpose, the cells were exposed to different concentrations of QS-21 and QA ranging from 0 to 1 × 10^3^ µM. Although both compounds had lytic effect on SNU1 and KATO III cells at the high concentration range (Fig. 3), their impact was not significant at the lower range of concentrations where MTS test revealed the loss of cell viability, as shown in Fig. 4. For example, the estimated viability value in terms of membrane integrity (as determined by the release of LDH) in SNU1 cells exposed to the IC_50_ doses for metabolic impairment, for QA and QS-21 were 78% and 72%, respectively, while for KATO III cells were 78% and 77%, respectively (Fig. 4). The release of significant amounts of LDH was observed only at higher concentrations of QA and QS-21 in both SNU1 and KATO III (> 100 µM), as shown in Figs. 3A, B and 4. This result showed that QS-21 and QA had a higher effect on SNU1 than KATO III cells. Therefore, we conclude that the cytotoxic effect (in terms of loss of cell viability determined by MTS) of QS-21 and QA was not exclusively related to their ability to disrupt membranes (as evidenced by the cell viability test based on the release of LDH). This finding was promising and led us to investigate if apoptotic mechanisms were involved in the observed loss of viability (as metabolic impairment) of both tumor cell types after exposure to middle to low doses of QS-21 and QA.

### Assessment of the effect of QS-21 and QA fractions on DNA damage and induction of caspases

**Figure 5:**
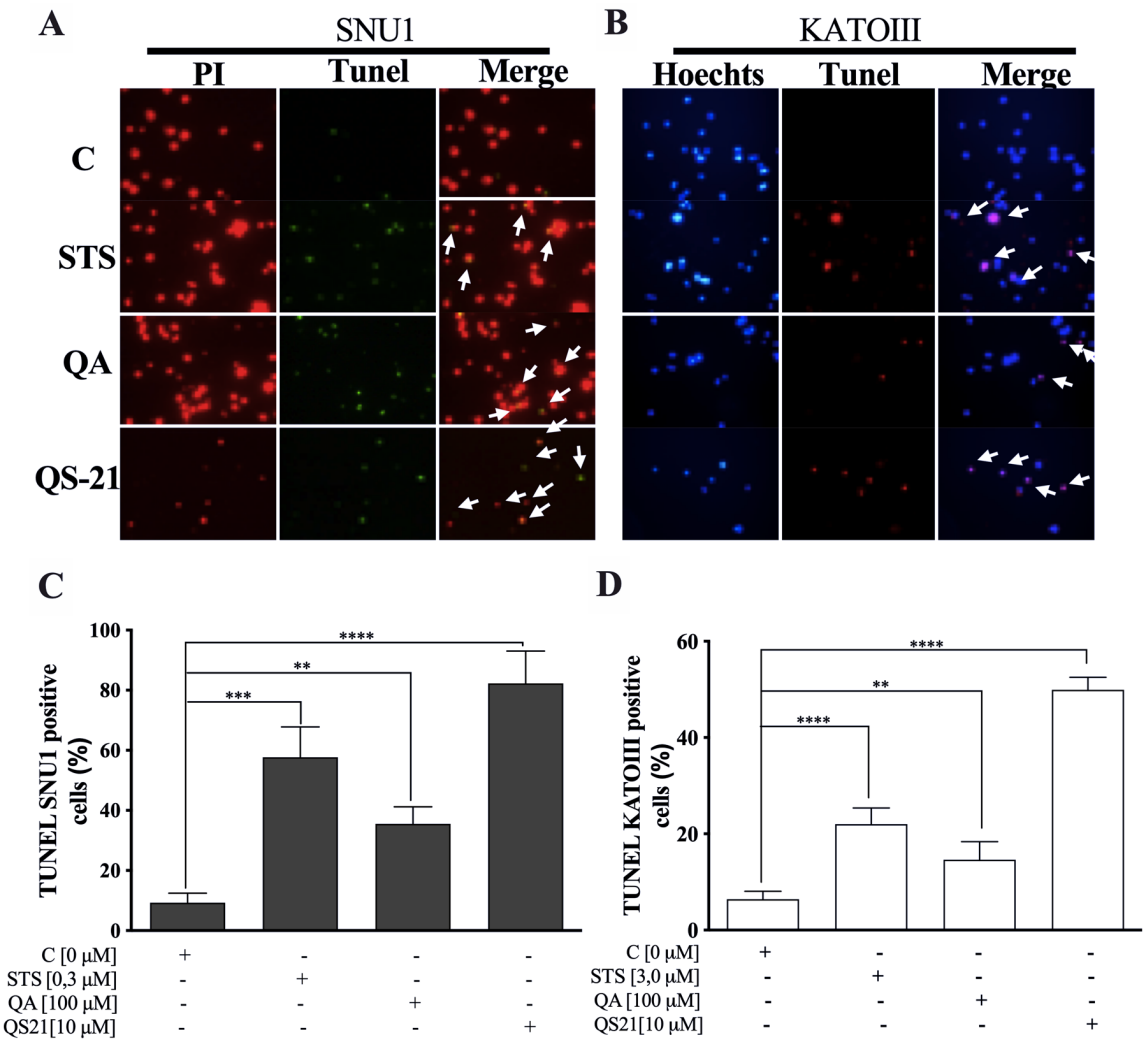
QS-21 and QA induce apoptosis by fragmentation of DNA (TUNEL assay) on SNU1 and KATO III. (**A**) and (**B**) are representative images of TUNEL staining. The TUNEL positive cells (bright green fluorescence, Fluorescein 12-dUTP) with DeadEnd Fluorometric TUNEL kit and TUNEL positive (red fluorescence) with Click-iT TUNEL Alexa Fluor 594, were observed after treatment of 1 × 10^5^ cells with 10 μM QS-21 and 100 μM QA, respectively. As negative control the cells untreated and positive control cells treated with 0.3 and 3.0 μM STS. All SNU1 cells were stained with PI (red fluorescence) and KATO III cells were stained with Hoechst 33342 (blue fluorescence). The white arrows indicate the apoptotic cells. (**C**) **P < 0.005, ***P < 0.0005 and ****P < 0.0001 SNU1 treated cell versus the control group; (**D**) **P < 0.005 and ****P < 0.0001 KATO III treated cell versus the control group. Eight fields were examined in each experiment using × 400 magnification and all the date are shown as mean ± SD of three separate experiments.

**Figure 6:**
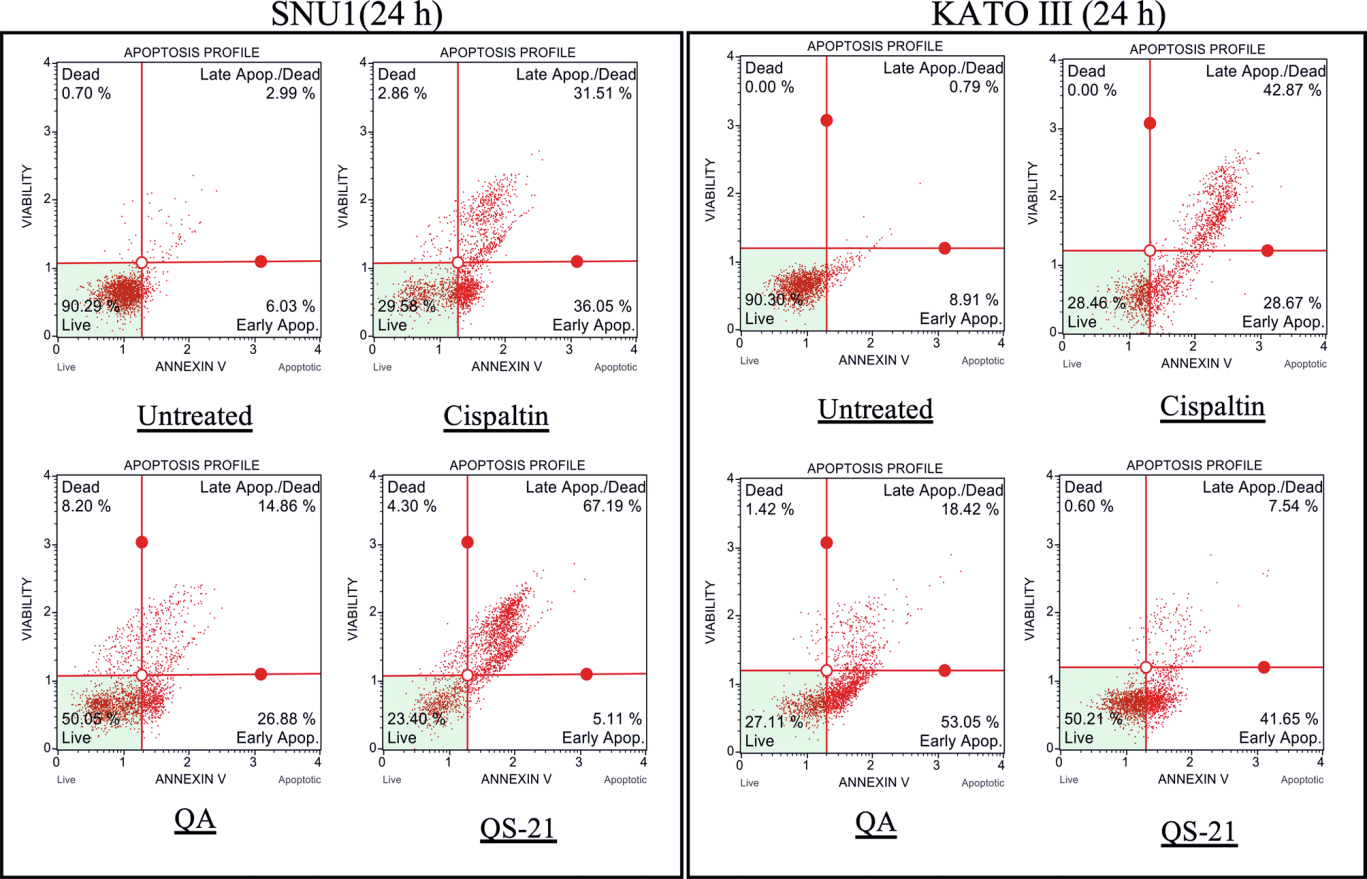
QS-21 and QA induce Apoptosis on SNU1 and KATO III. 1 × 10^5^ cells were treated with QS-21 (10 μM) or QA (100 μM) by 24 h and the distribution of the apoptotic cells was determined by flow cytometry. The percentage of early and late apoptotic cells (apoptosis rate) was estimated in comparison to the untreated cell and as positive control the cells were treated with Cisplatin (55 μM). SNU1 (left panel) and KATO III (right panel).

**Figure 7:**
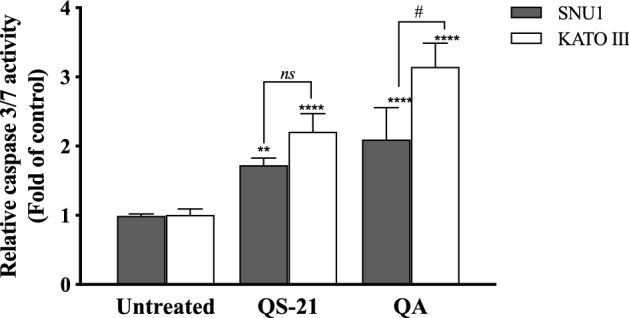
QS-21 and QA induce caspase 3/7 activity on SNU1 and KATO III. Approximately 2 × 10^4^ cells were treated with QS-21 (10 µM) or QA (100 μM) by 24 h. The Caspase-Glo 3/7 was measured showing that QS-21 and QA induced caspase 3/7 activity in both cell lines. Columns, mean (n = 5); bars, SD. ****or ^#^ *P* < 0.0001; ** *P* < 0.005 and *ns* not significant.

When SNU1 cells were incubated with 10 µM QS-21 for 24 h, was 82% (*P*<0.0001) of TUNEL positive cells compared with untreated cells as is shown in the Fig. 5A, C, while the incubation with 100 µM QA reached a 36% (*P* <0.005) of TUNEL positive cells (Fig. 5C, D). The effect of QA at 100 µM on KATO III cells resulted in low percentages of DNA damage reaching 15% (*P* < 0.005), as shown in Fig. 5D. The treatment of KATO III cells with QS-21 (10 μM) showed a prominent effect in the induction of cellular apoptosis, generating 50% (*P* < 0.0001) of TUNEL positive cells (Fig. 5B, D). Interestingly, we observed that QS-21 is more efficient than QA and STS to trigger the DNA fragmentation on SNU1 and KATO III. In order to confirm this result obtained by TUNEL assay, we employed flow cytometry using Annexin V and 7-Aminoactinomycin D. The apoptosis results are shown in Fig. 6, where the percentage of SNU1 cells undergoing apoptotic cell death increased to 42% and 72% for QA and QS-21, respectively, compared with untreated cells. In the case of KATO III after exposure to 100 μM of QA or 10 μM of QS-21 reached 73% and 49%, respectively (Fig. 6). Finally, we tested the induction of caspases 3/7 on both tumor cell lines with 10 µM of QS-21 and 100 µM of QA during 24 h, resulting in a significant induction of the activity of caspases 3/7 compared with the negative control (*P* < 0.0001) as seen in Fig. 7.

### In silico physicochemical characteristic and molecular docking

Table 2 showed the predicted binding energies (Δ*G*_*binding*_) for the compounds in complex with a set of cancer-related proteins with known 3D structure overexpressed in several cancer cell lines, SNU1 and KATO III cell lines. Among the mentioned proteins, both QS-21 and QA bind more strongly to pro-apoptotic protein BID (PDB entry: 2BID) than other proteins, with ΔG_binding_ values − 8.2 and − 9.9 kcal/mol, respectively.

**Table 2:** Binding free energies and in silico parameters of QS-21 and QA.

PDB entry Compd	∆G_binding_ (Kcal/mol)							Physicochemical parameters^h^	
	5CIR^a^	3CQW^b^	1F16^c^	2BID^d^	1E0O^e^	3EZQ^f^	3PZE^g^	LogD_7.2_	pKa1
QS21	− 6.7	− 7.5	− 6.3	− 8.2	− 6.6	− 5.5	− 7.5	− 6.92	3.28
QA	− 7.1	− 8.5	− 8.3	− 9.9	− 6.7	− 7.1	− 8.1	1.85	4.60

^a^Death receptor 4 (DR4), ^b^AKT1, ^c^pro-apoptotic protein BAX, ^d^pro-apoptotic protein BID, ^e^FGFR2, ^f^FAS protein and ^g^JNK1. ^h^In silico data were calculated using MarvinSketch software (version 18.24.0, ChemAxon Ltd.).

**Figure 8:**
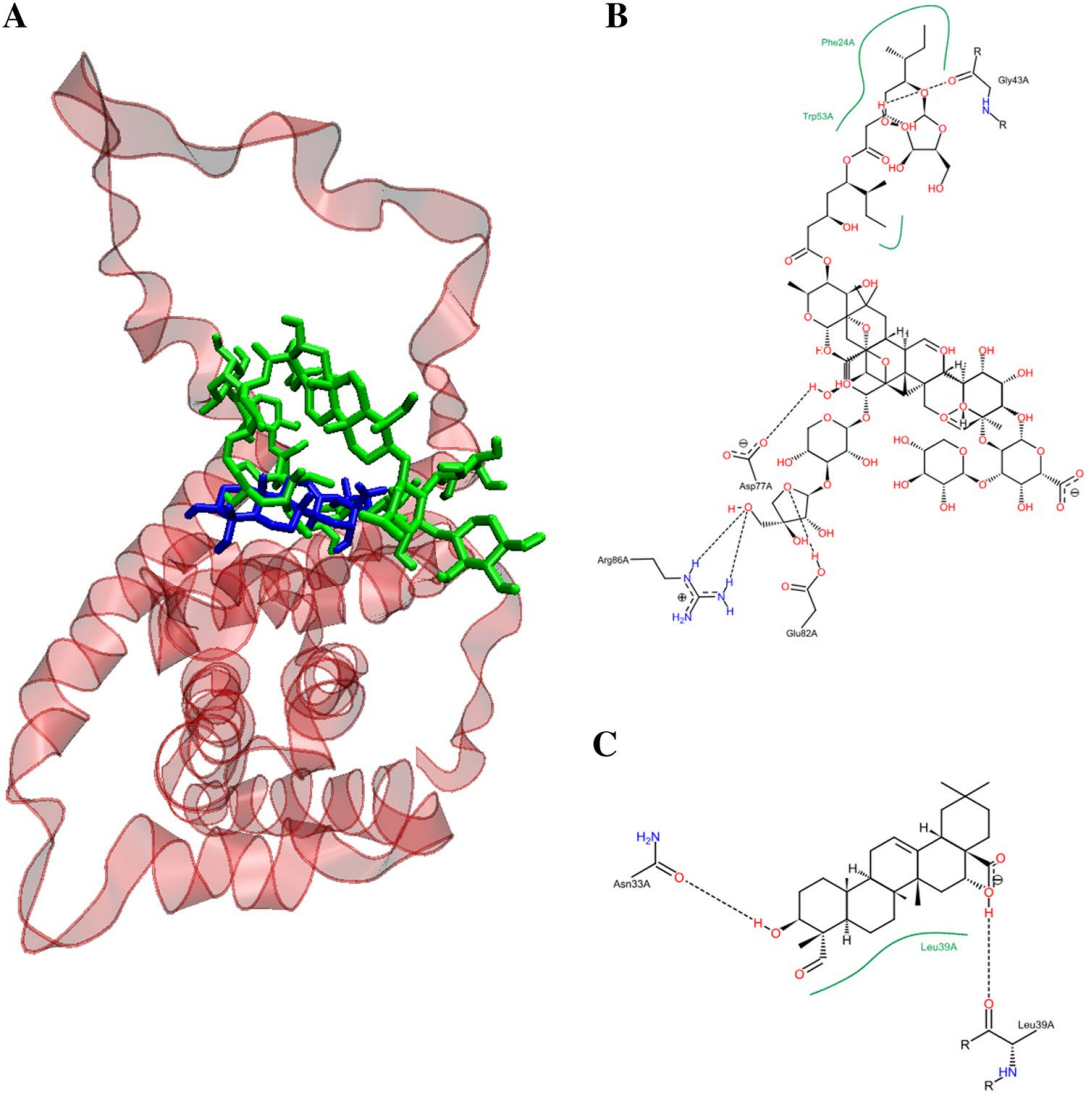
Potential binding site for the pose of lowest energy of docked QS-21 and QA within the pro-apoptotic protein BID (PDB entry: 2BID). (**A**) Ribbon representation of the 2BID with ligands inside the active site. QS-21 and QA are colored green and blue, respectively. A detailed inspection of the binding site within 2BID shows the amino acids responsible for the interaction with QS-21 (**B**) and QA (**C**).

Figure 8 depicts the potential binding site and pose of docked QS-21 and QA into 2BID, where it can be observed that both compounds lie in the same 2BID binding cavity. However, QS-21 and QA differ both in orientation and conformation in the binding site, leading to selective interaction of each compound with specific aminoacidic residues in the binding site. The interactions of each compound with the binding pocket of 2BID are governed mostly by hydrogen bonding and hydrophobic interactions. The amino acids responsible for the hydrogen bonding for 2BID with QS-21 are Gly43, Asp73, Glu82 and Arg86, whereas with QA are Asn33 and Leu39 (Fig. 8B). On the other hand, the amino acids responsible for hydrophobic contacts with QS-21 are Phe24 and Trp53, whereas with QA is Leu39 (Fig. 8C). Furthermore, as shown in Table 2, logD and pKa values were determined for the compounds. QS-21 and QA showed pKa values 3.28 and 4.60, respectively. These findings indicate that both compounds should be found mostly as their respective anionic forms at a physiological pH (~ 7.4). Furthermore, QS-21 and QA showed the LogD_7.2_ values − 6.92 and 1.85, respectively, indicating that QS-21 is a hydrophilic compound and QA is a hydrophobic compound.

## Discussion

In the present study, we showed that QS-21 and its aglycone QA from *Q. saponaria* exert cytotoxic effect in a dose-dependent against SNU1 and KATO III cell lines by MTS assay. Furthermore, QS-21 and QA have a better effect on SNU1 than KATO III cells. In addition, these compounds have a low effect on permeability of the cell membrane evaluated by LDH release assay. In fact, the LDH results indicate that the metabolic impairment occurred without causing loss in membrane integrity. Considering the above, we confirm, by TUNEL, Annexin V and caspase assay, that QS-21 and QA trigger an apoptosis process, where QS-21 was more efficient at inducing apoptosis on SNU1 than on KATO III.

According to the calculated hydrophobicity index, QA (LogP value of 1.85) could easily cross the cell membrane, whereas QS-21 is least likely due to its lower hydrophobicity index (LogP value of − 6.92). Therefore, these results (viability and LDH release) would be explaining why these compounds have a different response on SNU1 and KATO III cells. Indeed, studies have demonstrated that triterpenoid saponins and their aglycones are able to inhibit proliferation and to induce death by apoptosis on various cancer cell lines through lytic effect by permeabilization of membranes or by an interaction with the plasma membrane^12,13^. Evidence indicates that rearrangement of lipid rafts with Avicin D, another plant triterpenoid saponin, would be involved in cell survival or death^13–16^. Therefore, QS-21 could also trigger cell apoptosis through rearrangement of the lipid rafts on cell membrane or inducing trimerization of DR4 or FAS^16,17^. Docking results showed that QS-21bind more strongly to the DR4 receptor than FAS receptor, as shown in Table 2. Furthermore, our docking results also showed QS-21 and QA have better binding affinity to pro-apoptotic protein BID, therefore these compounds could be involved in the induction of apoptosis through of pro-apoptotic protein, besides caspases activation such as saikosaponin D in hepatocytes^17^. Interestingly, our docking studies show that QS-21 interact with the BH3 domain of pro-apoptotic protein BID, just like standard anticancer agents as venetoclax, obatoclax, navitoclax, and prodigiosin^18–20^. The BH3 domain is a conserved region in the Bcl-2 family members and critical for initiating apoptosis^21,22^. Although QA and QS-21 share the same binding pocket in protein BID, they differ both in orientation and conformation by selectively interacting with specific binding site residues, including amino acids responsible for hydrogen bonding (see Fig. 8), because both compounds have different molecular sizes due to sugars present in the structure of QS-21.

Our study have shown that QS-21 has cytotoxic effect on human gastric cancer cells, showing IC_50_ values around 7.4 µM for SNU1 and KATO III, almost 3 times better than previously studies with triterpenoid saponin extracted from the roots of *Adenophora triphylla var. japonica*^23,24^*.* This finding suggests that the presence of the acyl group at C28 position could provide a major cytotoxicity to the QS-21. While QA showed IC_50_ values for SNU1 and KATO III in a similar range (5–100 µM) to that reported with triterpenoid aglycones on different tumor cells lines^7,24–26^. Additionally, both compounds were selective against gastric tumor cells without affecting the proliferation of GES-1 cells, as shown in Fig. 2 and Table 1.

In conclusion, our findings suggest that QS-21 and its aglycone QA have significant in vitro antitumor selective activity against human gastric cancer cells, SNU1 and KATO III, inducing cell death by an apoptotic mechanism involving caspases activity and DNA fragmentation. However, in terms of the IC_50_ profiles obtained with both compounds, QS-21 demonstrated to be a more potent antitumor agent than QA. It is important to note that to confirm these in vitro and our docking studies, would be necessary to evaluate in a future the mechanism involved in the generation of cellular death, that is, if QS-21 induces the death cellular through of extrinsic or intrinsic pathway and to evaluate if QA have ability for across to cell membrane, triggering cell death. Additionally, it would be very interesting to evaluate the antiproliferative activity of QS-21 and QA on other cancer cell lines.

## Materials and methods

### Cell lines

The human GC cell lines (SNU1 and KATO III) were obtained from the American Type Culture Collection ATCC (Manassas, VA, USA) and were maintained in RPMI 1,640 supplemented with 10% FBS, penicillin 100 U/mL and streptomycin 100 μg/mL. The human gastric epithelial cell line (GES-1) was used as healthy control, maintained in DMEM medium (kindly donated by Dr. Dawit Kidane-Mulat from the University at Texas-Austin).

### Cell assays

MTS assay CellTiter 96 AQueous One Solution Proliferation Assay, Dead End Fluorometric TUNEL system and Caspase-Glo 3/7 assay purchased from Promega, (Madison, WI, USA). Click-it TUNEL Alexa Fluor 594 from Life Technologies. Muse Annexin V & Dead Cell Assay (Merck, Millipore, USA). LDH-Cytotoxicity Assay was purchased from Thermo Scientific (Thermo, Waltham, MA, USA). Staurosporine (STS), propidium iodide (PI) and Cisplatin were obtained from Sigma-Aldrich (St. Louis, MO, USA). The cell culture media (RPMI1640, DIMEN) and heat inactivated fetal bovine serum (FBS) as well as the antibiotics (penicillin and streptomycin) were purchased from Corning CellGro (New York, NY, USA).

### Triterpenic compounds

Samples of QS-21 and QA were supplied by Desert King International (CA, USA). QS-21 was purified from a highly purified extract of the bark of *Q. saponaria* (SuperSap, Desert King International). The extract was fractionated by preparative reverse phase HPLC column in an octadecylsilane column eluted with a gradient of acidified water and acetonitrile following a general approach described elsewhere 6. Fractions containing QS-21 were pooled together and lyophilized, rendering the QS-21 fraction employed in this study. QA was prepared by acid hydrolysis of the highly purified *Q. saponaria* extract (VaxSap, Desert King International, CA, USA), following a general procedure described elsewhere by Rodríguez-Díaz et al.^27^. For the biological testing of QS21 and QA, a stock solution of each compound in dimethyl-sulfoxide (DMSO) was prepared (2.5 mM and 0.1 mM, respectively).

### Testing of cytotoxic activity by MTS test

SNU1, KATO III and GES-1 (5 × 10^5^ cell/well) were seeded into a 96-well plate in the respective medium and incubated during 24 h at 37 °C in a humidified 5% CO_2_ atmosphere to recover the cells. Both compounds were tested by 24 h; QS-21 (0–50) µM on SNU1, KATO III, and GES-1 cells, respectively; QA (0–250) µM on SNU1, KATO III and GES-1 cells, respectively. STS, a protein kinase inhibitor, was used as positive control of death on SNU1 and KATO III cell lines determined by MTS assay, using concentrations described in the literature^28,29^ (Supplementary material S1). MTS assay was performed according to the manufacturer’s instructions. Then, the absorbance at 490 nm was recorded using an Epoch ELISA reader (ELx800, BioTek, VT, USA. Cells treated with DMSO 0.2% or PBS were included as vehicle control. The cell viability (expressed as percent of the viability of the cells not exposed to QS-21or QA). The experiment was performed in triplicate. The IC_50_ values for QS-21 and QA were determined.

### Evaluation of cytotoxic activity by the release of lactate dehydrogenase enzyme (LDH assay)

The cytotoxic effect of both triterpenic compounds on the cell membrane integrity was determined by detecting the release of LDH to the extracellular medium. 3 × 105 cells/well (SNU1 and KATO III) were seeded into a 96-well black plate with RPMI 1,640 as described above*.* Each triterpenic compound was added to the cell suspensions to the following concentrations; (1) QS-21, 0–12 µM; (2) QA, 0–125 µM. LDH Cytotoxicity Assay was performed according to the manufacturer’s instructions. The absorbance was measured at 490 nm and 680 nm using an Epoch ELISA reader (ELx800, BioTek, VT, USA a plate-reading spectrophotometer to determine LDH activity. Cytotoxicity was expressed in percentage. The experiment was performed in triplicate.

### TUNEL staining assay

1 × 10^5^ cells of SNU1 and KATO III were seeded into a 35 mm plate as described above. Then, the cells were supplemented with either QS-21 (10 µM), QA (100 µM) or STS (positive control of cellular apoptosis); the concentration of STS in each assay was 0.3 µM (SNU1) and 3.0 µM (KATO III), and further incubated during 24 h under the above conditions (Supplementary figure S1). SNU1 cells were analyzed using the Dead End Fluorometric TUNEL system kit according to the instructions of the manufacturer. The KATO III cells were analyzed using the kit Click-it TUNEL Alexa Fluor 594. The reason to use a different kit was due to high nonspecific background fluorescence with the first kit. Briefly, the incubated SNU1 and KATO III cells were fixed, permeabilized and labelled with fluorescein 12-dUTP and Alexa Fluor 594, respectively. This step was performed in a Nikon H550L fluorescence microscope equipped with a standard fluorescence filters set to: (1) 520 nm to view the apoptotic SNU1 cells labelled with Fluorescein-12-dUTP (green fluorescence); (2) 460 nm to visualize all SNU1 cells stained with Propidium Iodide (PI: red fluorescence); (3) 590 nm to view the apoptotic KATO III cells labelled with Alexa Fluor 594 (red fluorescence), and (4) 490 nm to visualize the blue fluorescence of all KATO III cells stained with Hoechst 33342. Cells were photographed in 8 different fields using 400X magnification.

### Annexin V assay

2 × 10^5^ cells of SNU1 and KATO III were seeded in 6-well plates as described above. Then, SNU1 and KATO III cells were supplemented with QS-21 (5 µM) or QA (100 µM). Cisplatin (55 µM), was used as positive control and untreated cells was the negative control. All the assays were incubated during 24 h. After incubation, the cells were washed with 1 mL PBS and 100 µL of Muse Annexin V& Dead Cell reagent were added. The apoptosis was measured using Muse cell analyzer and Muse analysis software (Merck-Merck Millipore) and cells were classified into four groups: live, early apoptotic, late apoptotic and dead or necrosis.

### Caspases activity assay

2 × 10^4^ cells (SNU1 and KATO III) were seeded in 96-well plates as described above. Then, the cells were supplemented with QS-21 (5 µM) or QA (100 µM), and incubated during 24 h. Activities of caspases 3 and 7 upon incubation of the cells were detected using the Caspase-Glo 3/7 assay following the manufacturer’s instructions. The fluorescence of the lysates (proportional to the amount of caspases activity present) was measured at 510 nm in an Appliskan multi-well fluorescence plate reader (Thermo Fischer Scientific, Waltham, MA, USA).

### In silico physochemical parameters prediction and molecular docking

The 2D structure of QA (CID: 101,810) and QS-21 (CID: 73,652,135) were retrieved in SDF file format from the PubChem database at NCBI (https://pubchem.ncbi.nlm.nih.gov). These molecules were visualized and their pKa and LogD values were calculated using Marvin Sketch software (version 18.24.0, ChemAxon Ltd.). Cl^−^ and Na^+^ K^+^ concentrations were set to 0.108 mol/L and 0.133 mol/L, respectively, for the calculation of logD. Tautomerization for QA and QS-21 were considered for the calculation of pKa and LogD.

We resorted to virtual screening using Autodock Vina, a target-specific scoring method useful for virtual screening^30^. QS-21 and QA were docked into a set of protein pockets to identify the target protein that could be inhibited potentially by these compounds. Here we performed rigid docking taking whole receptor in order to identify the potential binding pockets of proteins***. ***Crystal structure of proteins, including enzymes, growth factor, receptors and pro-apoptotic protein, were retrieved from the Protein Data Bank (PDB)^31,32^ using the PDB IDs 5CIR, 3CQW, 1F16, 2BID, 1E0O, 3EZQ and 3PZE (see more information in Table 2). Both ligands and proteins were prepared using AutoDock Tools version 1.5.6 (ADT), as previously described^32^. Finally, graphic analysis of molecular docking studies was performed using VMD^33^.

### Data processing and statistical analysis

Results were performed expressed as the means ± standard deviation (SD). Data was analyzed by two-way and one-way analysis (ANOVA) with Tukey's multiple comparisons test (*P* < 0.005) using GraphPad Prism 8 (GraphPad Software, San Diego, CA, USA).

## Supplementary information

Supplementary file
