# Supplementary material for: In vitro evaluation and molecular docking of QS-21 and quillaic acid from Quillaja saponaria Molina as gastric cancer agents
Source: Sci Rep. 2020 Jun 29;10:10534. doi: 10.1038/s41598-020-67442-3 (PMC7324585; doi:10.1038/s41598-020-67442-3)
Supplement: Supplementary file 1 — Supplementary file [file 41598_2020_67442_MOESM1_ESM.pdf]

Supplementary Figure S1

***In vitro* evaluation and molecular docking of QS-21 and quillaic acid from *Quillaja saponaria* Molina as gastric cancer agents**. Leda Guzmán, Katherine Villalón, María José Marchant, María Elena Tarnok, Pilar Cárdenas, Gisela Aquea, Waldo Acevedo, Leandro Padilla, Giuliano Bernal, Aurora Molinari and Alejandro Corvalán.

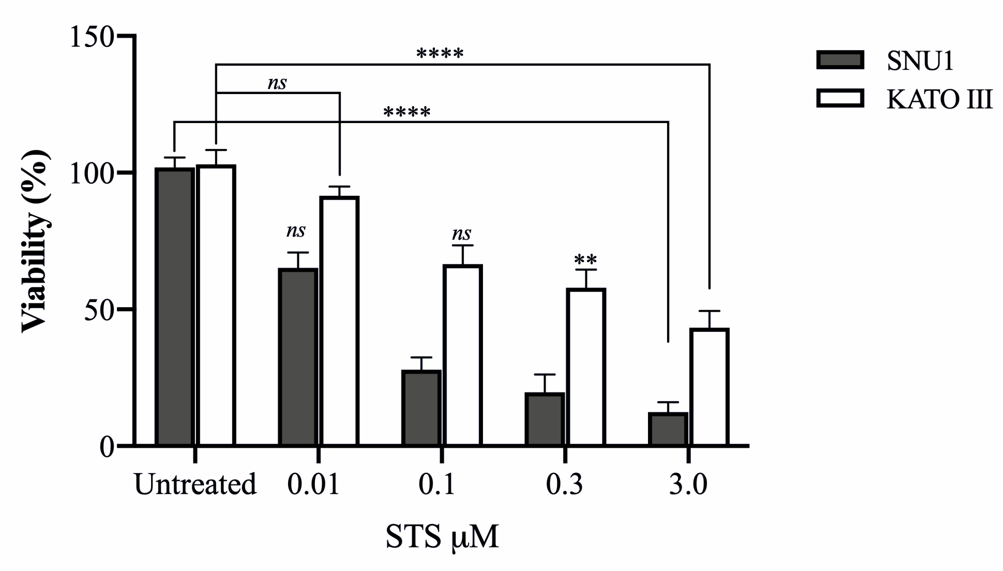

***Figure S1. Effect of Staurosporine on cell viability**. Kato III, SNU1 (≈5×10^5) were treated with different concentrations of STS (0.01-0.1-0.3 µM) for 24 h. Cell viability was measured using MTS assay. Data expressed as means ± SD of three independent experiment. ****$P < 0.0001$ SNU1 and KATO *vs* untreated, *ns* (no significant) between SNU1 0.01 µM *vs* KATO III 0.01 µM, ** $P < 0.005$ KATO III 0.03 µM *vs* KATO III 3.0 µM.

*Accordingly, to apoptotic assay was considered 0.3 µM of STS as a positive control to SNU 1 with a viability of 25.51% ± 15.40 and 3.0 µM to KATO III with the viability of 41.92% ± 5.2.
